# Centering the Right to Health of Childbearing People in the US During the COVID-19 Pandemic

**DOI:** 10.3389/fpubh.2022.862454

**Published:** 2022-06-02

**Authors:** Cecília Tomori, Bhavana Penta, Rebecca Richman

**Affiliations:** ^1^Johns Hopkins University School of Nursing, Johns Hopkins University, Baltimore, MD, United States; ^2^Johns Hopkins Bloomberg School of Public Health, Johns Hopkins University, Baltimore, MD, United States

**Keywords:** COVID - 19, childbearing people, pregnancy, birth lactation, human rights, equity, reproductive health, pandemic policy response

## Abstract

Childbearing people in the US have experienced the double burden of increased risks from infection and significant disruptions to access and quality of essential health care services during the COVID pandemic. A single person could face multiple impacts across the course of their reproductive trajectory. We highlight how failure to prioritize this population in the COVID-19 policy response have led to profound disruptions from contraception services to vaccination access, which violate foundational principles of public health, human rights and perpetuate inequities. These disruptions continued through the omicron surge, during which many health systems became overwhelmed and re-imposed earlier restrictions. We argue that an integrated pandemic response that prioritizes the healthcare needs and rights of childbearing people must be implemented to avoid deepening inequities in this and future pandemics.

## Introduction

Health care systems globally experienced profound disruptions from the COVID-19 pandemic. The US has experienced exceptionally high infection and death toll due to inadequate and inconsistent implementation of public health measures. This response has simultaneously left childbearing people and young children vulnerable to infection, while also exposing them to significant disruptions in healthcare. The pandemic response has resulted in violations of the framework of respectful maternity care which at its core includes human rights including the right to health, right to information, non-discrimination, and benefits of scientific progress (1, 2). In this perspective we highlight some of these disruptions that violate human rights and perpetuate inequities. These selections are not intended as a comprehensive review, but rather serve to highlight the pervasive nature of this problem across the reproductive spectrum. We argue that this vulnerable population's rights and well-being must be brought to the forefront and map out a path forward that centers these rights.

### Restrictions on Contraceptive & Abortion Access

The classification of certain procedures related to contraceptive care and abortion access as elective or non-essential during the COVID-19 pandemic has had serious repercussions across the reproductive spectrum. Barriers surrounding insertion and removal of long-acting reversible contraceptives (LARC) and tubal ligation procedures infringe upon an individual's right to choose when to conceive and disproportionately impacts marginalized communities (3). A Guttmacher survey found that Black and Hispanic women disproportionately experienced barriers to birth control access (38 & 45%) compared with their White counterparts (29%) (4).

Pandemic protocols aiming to control infection were actively exploited to restrict abortion access. In March 2020, eleven conservative governors decided to omit abortion care in the list of essential services, thus making it inaccessible for women across the nation (5). Under the justification of rationing personal protective equipment (PPE), these elected officials limited a fundamental service that often requires few resources. Many medical experts and professional societies expressed concern that these restrictions were driven by political agendas rather than a consideration of public health and reiterated that medication abortions require little to no PPE (6). Those seeking abortion services in these states are not only required to travel greater distances, but likely continue to face additional economic hardship related to the realities of COVID-19 (7). Between the months of March and April, 2020 there was a 706% increase in Texan patients accessing services in surrounding states such as Colorado, Mexico, and Nevada (8). Service delivery disparities across states continue to proliferate. For instance, the FDA mandate to remove the in-person requirement for mifepristone administration (medication abortion) (9) expanded access in many states, but 19 states currently ban telehealth abortions (9), ultimately leading to more pronounced inequities.

### Impacts on Fertility & Prenatal Care

The early categorization of non-emergency fertility care as a non-essential service was backed by the American Society of Reproductive Medicine (ASRM) based on the risks posed by frequent visits and personnel needs (10). While the rationale behind pausing services was appropriate, it was associated with significant psychological distress for women experiencing delay in fertility care (11).

The abrupt transition to telehealth appointments for prenatal services during the initial phases of COVID-19 was similarly implemented to reduce viral transmission. Recent studies have demonstrated overall patient and provider satisfaction with telehealth prenatal care (12, 13). However, overall satisfaction can conceal inequities including technological barriers, unreliable data connection, and difficulties utilizing interpreter services (14). These concerns further perpetuate existing health disparities, particularly for women with limited English proficiency, those living in rural areas where internet access is limited or for those unable to afford these services (13).

### Restrictions of Perinatal Support

The global pandemic has further compounded existing severe racial inequities in birth outcomes, wherein Black women and infants face significantly higher mortality and morbidity than their White counterparts (15). Birth doulas, who provide culturally appropriate, continuous labor support, are critical in fighting these racial inequities (7). Despite this evidence and statements from professional societies about the importance of doulas as essential care providers, many hospitals limited their presence in the delivery room in the first wave (16), and restrictions have been re-imposed in subsequent surges. Hospital policies have varied widely from only allowing the partner, choosing between a partner and doula, or offering support for virtual doula connection during delivery (16). Policies also varied at the state level; New York, New Jersey, and Michigan were initially among the few states with explicit executive orders advocating for doulas to be considered separately from support persons (17).

Doula accessibility is particularly important for women of color and women with limited English proficiency (LEP). Restrictions for in-person interpretation and culturally competent doulas have led to further disparities among Latina women in particular (18). While some institutions facilitated connections to outside doula support, other healthcare settings restricted even video calls during delivery (19). The challenges faced by community-based doula organizations to transition to virtual services and the lack of technological access among mothers continue to hinder virtual doula support, and compound existing inequities (20). Moreover, Combellick et al. (21) have documented lack of clear information from healthcare providers and unnecessary interventions during the pandemic. However, access to doula- or midwifery support could counteract some of these challenges, thereby highlighting the importance of perinatal support and advocacy.

### Restrictions on Visitation During Delivery

During the early months of the COVID-19 pandemic, public outcry ensued after several health care facilities in New York restricted any visitors from being present during childbirth (17). While this regulation was swiftly addressed by Governor Cuomo's executive order, which prohibited the restriction of a support person during labor, the discussion regarding who can attend delivery remains an area of concern and debate. Arora and colleagues (17) have argued that hospital policies need to consider the principle of non-maleficence not only for healthcare professionals in relation to public good, but also for the vulnerable mother-infant dyad. During a period of intense emotional and physical stress, birthing persons should have access to a support person to weigh-in on decision-making and advocate for the client as needed (22). Despite these early concerns, an analysis of hospital policies throughout the US found that 66% of hospitals in the study only allowed one visitor, 23% allowed two visitors, and the remaining 11% worked on a case-by-case basis or had no established protocol (23). These restrictions have often been re-implemented in recent surges, threatening childbearing people's right to support.

### Impacts of Post-partum Separation Policies and Breastfeeding

Early 2020 CDC and American Academy of Pediatrics (AAP) guidance included mother-infant separation before it was reversed in summer 2020. Moreover, the guidance provided limited information about supporting human milk expression during separation or how mothers could effectively return from expression to feeding at the breast.

This guidance went against World Health Organization (WHO) recommendations that the mother-infant dyad should be kept together regardless of COVID-19 status if neither of them requires intensive care (24). Skin-to-skin (S2S) contact plays a pivotal role in infant health even among premature and sick infants (25). S2S is particularly crucial in the initiation of breastfeeding, and has both short- and long-term health consequences (24). Interfering with breastfeeding limits immunological protection from infection. Breastfeeding confers protection both through general anti-infective properties and specific antibodies once the mother encounters a pathogen or after vaccination (26). The lasting harms of separation and hindering breastfeeding are particularly concerning for marginalized communities who already have lower breastfeeding prevalence and higher infant mortality due to structural racism (25). While separation is no longer recommended, there are recent reports of separation during the omicron surge, and further impacts for mothers whose neonates require care in the NICU (27).

### COVID-19 Vaccination Access

Pregnant and lactating people have historically been excluded from vaccine trials and drug trials due to concerns for risk to the well-being of the fetus and growing infant. As a result, pregnant and lactating people were not included in the vaccine trials for the COVID-19 vaccine. While the vaccine was approved for emergency use by the FDA on December 11th, 2020, Pfizer only announced clinical trials in pregnant and lactating people on February 18th, 2021 (28). Per respectful maternity care guidance, childbearing people should be able to benefit from scientific progress and allowed participation. The lack of inclusion in the initial trials violates this very right (2). The CDC, the American College of Obstetrics and Gynecology (ACOG) and the Academy of Breastfeeding Medicine (ABM) recommended that the vaccine should not be withheld from pregnant or lactating people and encouraged individuals to engage in discussions with their primary healthcare provider regarding vaccination. Unfortunately, these discussions were based on limited evidence due to lack of trials and insufficient immunological knowledge on how vaccination would provide immunity to the dyad.

On July 30th, 2021, ACOG and CDC revised their stance and fully recommended vaccination based on an evidence of a robust vaccine response in this population without safety concerns (29, 30). Despite a September 2021 advisory strengthening this recommendation (31), as of April 30, 2022, nearly 30% of pregnant women overall, and 43% of Black pregnant women remain unvaccinated with additional inequities in booster uptake (32). Because vaccinations protect against poor maternal and neonatal outcomes (33), vaccination inequities worsen outcomes for these populations. Additionally, vaccination transfers immunity during pregnancy and antibodies during lactation (26, 29), respectively, which protects infants who are ineligible for the COVID-19 vaccines themselves.

## Discussion

### Lessons for Pandemic Preparedness Policies

Early in the COVID-19 pandemic, it became evident that both infection with SARS-CoV-2 and pandemic policy responses had a significant impact on the health of those seeking sexual and reproductive health services and their newborns. Policies directed at pregnant and lactating women and their newborns were particularly fragmented and inequities were overlooked. Yet, in the US the failure to prioritize these vulnerable groups in accordance with WHO guidance violated human rights and perpetuated existing inequities. Pandemic preparedness efforts should consider these impacts. The WHO's recommendations protect sexual and reproductive rights and health, and include well-developed guidance on maternity care and respectful birth (1, 2). Furthermore, the WHO highlights the life-saving effects of maternal-infant contact and breastfeeding, which is foundational to breastfeeding guidance and holds special significance in emergencies. A similarly integrated approach should be implemented for pandemic policies across the span of reproductive health and maternity care in the US. Consistent policies across states and health systems must be implemented that ensure equitable access to reproductive and perinatal services and support during birth, keep mothers and infants together whenever possible, protect breastfeeding and ensure timely access to vaccination. Moreover, expectant mothers should be supported in their rights to multiple birthing options (21), clear communication from healthcare providers (21, 34) and should not be subjected to unnecessary interventions (21). Midwifery-led care serves as a model to counteract the loss of humanized care and rights during the pandemic (21).

The right to health provides a normative foundation for emergency responses – focusing on state obligations to provide timely, accessible, and quality care and prioritizes the needs of those who are most marginalized (35). To date, US pandemic policy has not adequately drawn on this framework. Consequently, those seeking reproductive services, and particularly pregnant, lactating people and their infants from marginalized populations, have experienced violations of their rights and deepening inequities.

Policy development and implementation should incorporate a wide range of expertise, including human rights and equity experts and those receiving reproductive care themselves. These lessons require immediate attention in responding to emerging variants and future pandemics. As policies continue to evolve, maintaining and advancing the human rights of childbearing people must be a highest priority.

## Data Availability Statement

Publicly available datasets were analyzed in this study. These data can be found here: publications included in this Perspective are cited in References.

## Author Contributions

CT conceived the paper with input from BP and RR. All authors participated in reviewing the literature, drafting the manuscript, and revising the manuscript for publication.

## Conflict of Interest

The authors declare that the research was conducted in the absence of any commercial or financial relationships that could be construed as a potential conflict of interest.

## Publisher's Note

All claims expressed in this article are solely those of the authors and do not necessarily represent those of their affiliated organizations, or those of the publisher, the editors and the reviewers. Any product that may be evaluated in this article, or claim that may be made by its manufacturer, is not guaranteed or endorsed by the publisher.
